# The Fabrication and Mechanism of a Crystalline Organic Fluorescent Probe Based on Photoinduced Electron Transfer

**DOI:** 10.3390/molecules28196774

**Published:** 2023-09-23

**Authors:** Xinxin Zhang, Wei Liu, Mei Yang, Zhongyue Li

**Affiliations:** 1 School of Environmental and Material Engineering, Yantai University, Yantai 264005, China; zhangxinxin@s.ytu.edu.cn (X.Z.); meiyang@ytu.edu.cn (M.Y.); 2 School of Mechanical & Electrical Engineering, Henan University of Technology, Zhengzhou 450001, China; weiliuww@163.com

**Keywords:** crystalline structure, fluorescence detection, luminescence materials

## Abstract

The response performances of the crystalline organic fluorescence probe are highly dependent on the long-range ordered arrangement of crystalline structure. Herein, a novel organic crystalline fluorescent probe with a high quantum yield was established through the rapid self-assembly of 1,2,4,5-Tetrakis (4-carboxyphenyl) benzene (H_4_TCPB) and DMF molecules. Each H_4_TCPB, which connects to four DMF molecules through hydrogen bonds, acts as the structural unit. The building units are packed by π–π, lone pair···π, and lone pair···lone pair interactions to form solid-state crystalline materials. H_4_TCPB·4DMF exhibits distinct blue fluorescent under UV light, while the quantum yield is as high as 89.02% and the fluorescence lifetime is 1.95 ns. The H_4_TCPB·4DMF nanocrystal exhibits a specific fluorescence quench sensibility to tetracycline (TC), compared with the common chemicals and ions in environmental water. Moreover, the test results can be obtained quickly and are easily visible to the naked eye. The limit of detection for TC is as low as 12 nM in an aqueous solution. Spectral analysis and density functional theory (DFT) theoretical calculations were used to explain the fluorescence quenching mechanism of H_4_TCPB·4DMF toward TC, which could be attributed to the photoinduced electron transfer occurring from H_4_TCPB·4DMF to TC. Our work enriches the database of crystalline luminescent materials and provides theoretical support for the design and mechanical studies of organic fluorescent probes.

## 1. Introduction

Fluorescent materials have attracted significant attention owing to their great potential in optoelectronic science, fluorescence sensing, bioimaging, and environmental monitoring [1,2,3,4]. Fluorescence detection is widely used to detect environmental pollutants due to its high sensitivity, rapid detection, easy operation, and low cost [5,6]. Photoinduced electron transfer (PET) plays a significant role in fluorescence detection and many other photochemical scenarios. Particularly, PET, which is the process of electron transfer through photoexcitation, is considered to be responsible for fluorescence quenching in the field of fluorescence detection. Chemists have made great efforts to design, synthesize, and develop many luminescent materials for practical application and have made great progress. For instance, the concept of aggregation-induced emission (AIE) materials proposed by Tang and coworkers [7,8], in which molecular aggregates exhibit stronger emission than single molecules. Many new sensors have been developed based on the photoluminescence properties of organic functional molecules. They are usually manufactured from organic or inorganic photoluminescent materials. Compared to inorganic photoluminescent materials, which are often composed of toxic heavy metals or rare earth elements, nontoxic organic photoluminescent fluorescent probes have a more comprehensive range of application scenarios [9,10]. Organic fluorescence probes include crystalline organic fluorescence probes and amorphous organic fluorescence probes. They have unique physical–chemical properties due to the diversity of organic ligands and rich functional groups. The long-range ordered arrangement of crystalline organic fluorescent probes restricts intramolecular rotation and enhances the emission [11], facilitating specific and selective identification and the modification of their fluorescence properties when interacting with captured analytes. Solid-state crystal luminescent materials can generate different responses via different stimuli, including light [12], vapor [13], pH [14], and temperature [15], which do not change the chemical structure of the material. The detected substance may interact with the probe physically/chemically, resulting in a change in its luminescence state, while the change in fluorescence intensity (a decrease or increase) is one of the most easily observed phenomena in most sensing studies [16,17].

Tetracycline (TC) is a broad-spectrum antibiotic that is used to treat bacterial infections because of its low price and powerful function [18,19,20]. At present, TC is widely used in animal husbandry and aquaculture, resulting in a high level of residual TC in soil and water environments [21,22]. Moreover, accumulated TC enters the human body through the food chain and even endangers health in severe cases [23]. Therefore, it is necessary to detect and analyze the trace tetracycline in the sample quickly and selectively. Traditionally, such tests have been performed using large or expensive techniques such as mass spectrometry (LC-MS), high-performance liquid chromatography (HPLC), capillary electrophoresis (CE), and surface-enhanced Raman scattering (SERS) [24,25,26,27]. These kinds of methods are complicated and expensive, and their results are not immediately available. Hence, it is of great significance to develop a method with low cost, simple operation, low detection limit, noticeable, and rapid results.

**Figure 1:**
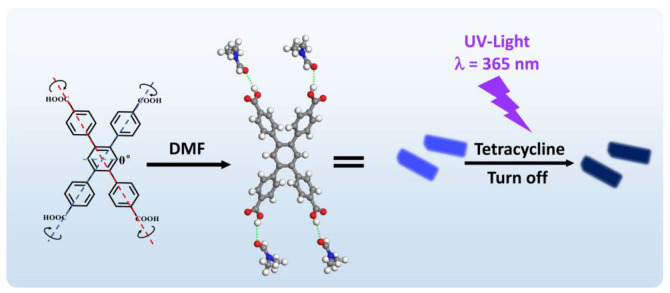
Schematic diagrams of the structure of H_4_TCPB·4DMF and the fluorescence quenching response toward tetracycline (TC).

In this work, we prepared a nanocrystalline organic fluorescent probe using an organic molecule, 1,2,4,5-Tetrakis (4-carboxyphenyl) benzene (H_4_TCPB), based on a solvent temperature-controlled rapidly crystallized method for detecting TC in water via a photoinduced electron transfer (PET) fluorescence quenching mechanism. H_4_TCPB has five benzene rings, one at the center and four on the side, resulting from the benzene ring and the relatively fixed X-shaped space stretch; it is partially flexible as the angle between the central benzene ring and the side benzene rings are rotatable before crystallization (Figure 1). H_4_TCPB and DMF were cocrystallized in a solvent to form molecular crystals of H_4_TCPB·4DMF. The bulk crystal was synthesized to investigate the microstructure of the molecular crystals, and the nanosized crystals were prepared using a rapid method to develop the nanofluorescence probe. Hydrogen bonding, π–π, lone pair···π, and lone pair···lone pair interactions were confirmed in the H_4_TCPB·4DMF crystal structure. H_4_TCPB·4DMF presents a significant blue fluorescence under UV light and shows a significant quenching effect by TC compared with other irons and chemicals. Hence, a rapid fluorescence detection method for TC was developed and the detection performances, including selectivity and sensitivity, were evaluated. Moreover, the sensing mechanism was investigated by combining spectral analysis and density functional theory (DFT) simulation.

## 2. Results and Discussion

### 2.1. Crystalline Structure of H_4_TCPB·4DMF

**Figure 2:**
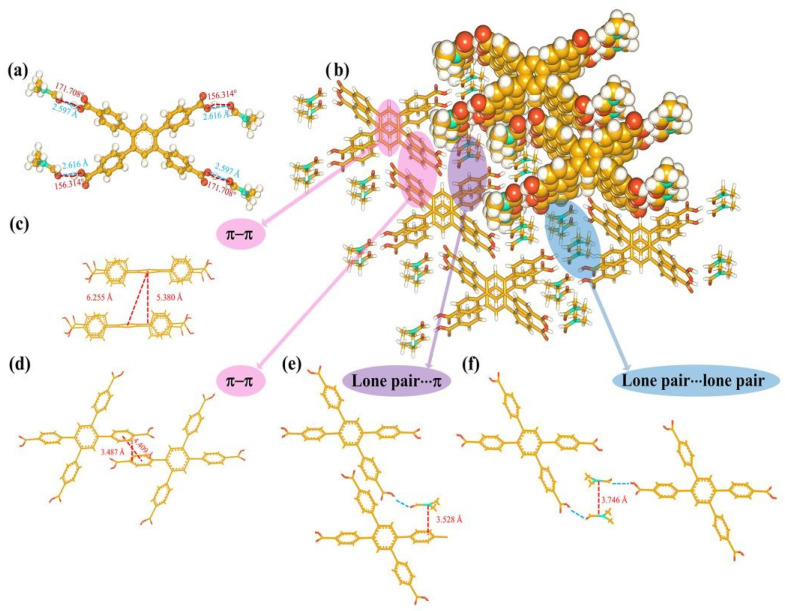
Crystal structure of H_4_TCPB·4DMF: (**a**) bond lengths and bond angles of hydrogen bonds between H_4_TCPB and DMF molecules; (**b**) stacking view of H_4_TCPB·4DMF along the (100) direction; (**c**) the π–π interaction of center benzene rings; (**d**) the π–π interaction of benzene rings on the side; (**e**) schematic representing the modes of lone pair···π contacts; (**f**) schematic representing the modes of lone pair···lone pair.

The single-crystal X-ray diffraction data of H_4_TCPB·4DMF have been mentioned in our previous work (HOF-TCPB-298) [28]. Here, they are described in more detail. The structure of the H_4_TCPB·4DMF molecule consists of five benzene rings and four carboxyl groups. One benzene ring is in the center, and four attached carboxyl phenyl groups can theoretically serve as the hydrogen bond donors. Also, in the crystal structure of H_4_TCPB·4DMF, each H_4_TCPB connected with four DMF molecules by hydrogen bonding (Figure 2a) which is considered to be a structure unit. The hydrogen-bonding distances between the two O atoms (d_O−H···O_) are 2.616 Å and 2.597 Å, and the corresponding angles (θ_O−H···O_) are 156.314° and 171.708°, which confirms the substantial hydrogen bonds. Such structure units are packed through π–π interaction and hydrogen bonds. The stacking view along the (100) direction is shown in Figure 2b, where some interactions are marked. As shown in Figure 2c,d, the distance between the rings in locations where π–π stacking interactions occured was calculated. The plane distance between phenyl rings at the center of each H_4_TCPB molecule is 5.380 Å, and the center distance of the phenyl ring is 6.255 Å. Moreover, the diagonally positioned phenyl rings of H_4_TCPB are located in the same plane, and the phenyl rings are in the same position as adjacent H_4_TCPB linkers. The distance between the center of the corresponding phenyl rings is 4.409 Å, while the distance between planes is only 3.487 Å. Figure 2e presents the lone pair···π contact between the nitrogen atom of the DMF molecule, which was linked to H_4_TCPB through hydrogen bonds, and the benzene ring. The distance between the nitrogen atom and the plane of the benzene ring is 3.528 Å. Moreover, there are lone pair···lone pair contacts between the nitrogen atoms in adjacent DMF molecules and the distance between them is 3.746 Å (Figure 2f). Based on these interactions, the building units of H_4_TCPB·4DMF stack and grow to the long-range ordered crystal materials.

### 2.2. Characterization of H_4_TCPB·4DMF

**Figure 3:**
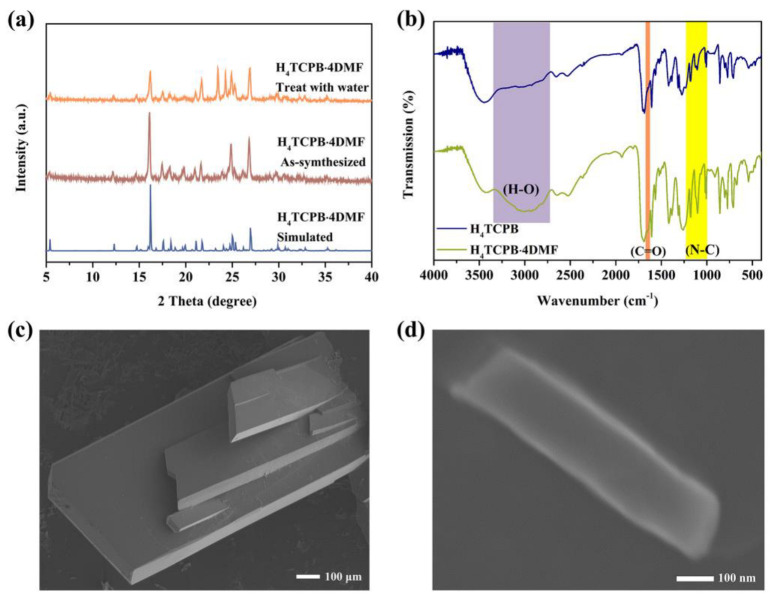
Characterizations of H_4_TCPB·4DMF: (**a**) FT−IR spectra; (**b**) PXRD patterns; (**c**) SEM image for the bulk crystals; (**d**) SEM image for the nanocrystal.

The experimental PXRD patterns of both the bulk crystal and nanocrystal of H_4_TCPB·4DMF are consistent with the simulated ones, indicating that the crystalline phase is pure. Moreover, the broadening of the PXRD peaks of the nanocrystal could be attributed to the small size effect (Figure 3a). The FT−IR spectra of H_4_TCPB and H_4_TCPB·4DMF were measured, and the results are displayed in Figure 3b. The peaks at 1650, 1178, 1103, and 1007 cm^−1^ are significantly enhanced in the FT−IR spectra of H_4_TCPB·4DMF compared to H_4_TCPB, which can be attributed to the C=O and C–N bonds of DMF. The regular arrangement of the highly crystallized H_4_TCPB·4DMF results in a significant enhancement of the broadband representing O–H belonging to the carboxyl located at 2700−3300 cm^−1^. The SEM images for both the bulk crystal and nanocrystal of H_4_TCPB·4DMF present rectangular sheets (Figure 3c,d). The bulk single crystal exhibits micron-size flake morphology, while the plan view size of the nanocrystalline is about 200 nm × 800 nm. The TGA of H4TCPB·4DMF was carried out in an argon atmosphere. As shown in Figure S1, H_4_TCPB·4DMF does not lose weight below 100 °C; with further heating, the hydrogen bonding between DMF molecules and H_4_TCPB will be gradually destroyed, and a significant weight loss can be observed owing to the departure of DMF; when the temperature reaches above 400 °C, H_4_TCPB begins to decompose.

### 2.3. Fluorescence Properties of H_4_TCPB·4DMF

**Figure 4:**
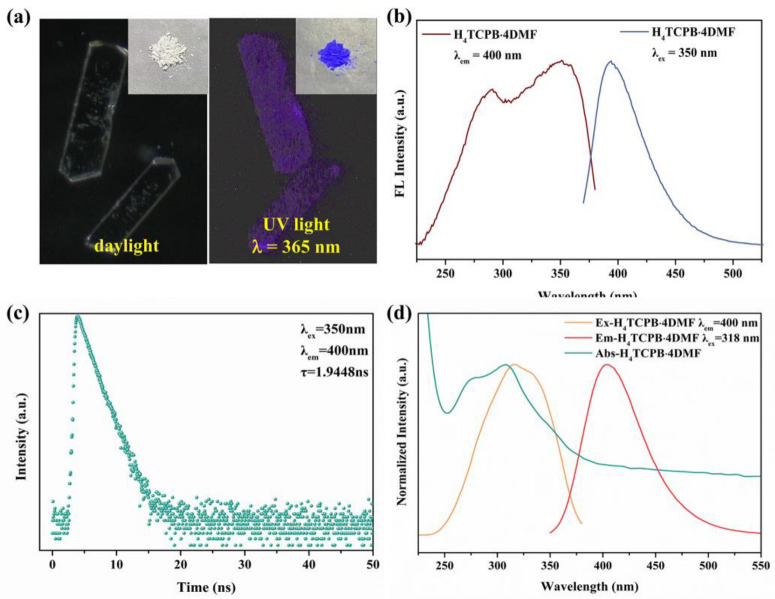
Fluorescence characteristics of H_4_TCPB·4DMF: (**a**) optical photographs of bulk crystal and nanocrystal samples (inset) under daylight and UV light of 365 nm; (**b**) solid fluorescence spectrum of nanocrystalline H_4_TCPB·4DMF; (**c**) decay curve of nanocrystalline H_4_TCPB·4DMF; (**d**) fluorescence and absorbance spectra of nanocrystalline H_4_TCPB·4DMF in aqueous suspension.

The fluorescence properties of H_4_TCPB·4DMF were investigated at room temperature. As shown in the optical photos of the bulk crystals and the nanocrystal sample (Figure 4a), the changes in the color of H_4_TCPB·4DMF under different lights are instantly visible to the naked eye. It is colorless in daylight and blue-purple in UV light (λ = 365 nm). This indicates that H_4_TCPB·4DMF can undergo fluorescence excitation with UV light, which should be attributed to the fact that the long range ordered arrangement in the crystal structure limits the rotation and vibration of the benzene rings, thus reducing nonradiative transition and providing better quantum yield [29]. This was further investigated using fluorescence spectral analysis. As shown in Figure 4b, the solid-state sample of H_4_TCPB·4DMF shows a width excitation band from 250 nm to 375 nm with a peak at 350 nm, and the emission spectra with the excitation wavelength of 350 nm peak at 400 nm. The fluorescence emission lifetime of the solid sample is τ = 1.9448 ns (Figure 4c), which exhibits the fluorescence behavior of H_4_TCPB·4DMF. The quantum yield is as high as 89.02% (Figure S2). Furthermore, the ultraviolet absorption, fluorescence excitation, and emission spectral analysis of the H_4_TCPB·4DMF aqueous suspension were performed (Figure 4d). The wavelength coverage of fluorescence excitation is consistent with that of ultraviolet absorption. The peaks of the fluorescence excitation and emission spectra are 318 nm and 400 nm, respectively.

### 2.4. Sensing of TC in Water

The flexible torsion between the benzene rings; a mass of hydrogen bonds; and π–π, lone pair···π, and lone pair···lone pair interactions, as well as the highly ordered crystalline state and its own photochemical properties, encourage us to develop H_4_TCPB·4DMF nanocrystals as fluorescence probes [16]. At the same concentration, when comparing different solvents, it can be seen that the fluorescence intensity of H_4_TCPB·4DMF in water is the strongest, and luminescence quenching can be observed by the naked eye under a laboratory 365 nm UV lamp, resulting in the most apparent quenching effect (Figure S3). The photoluminescence intensity of the suspension has a direct association with both aqueous solution and polar organic solvents [30]. The largest PL intensity in H_2_O can be attributed to the highest polarity. Moreover, using water as the dispersing agent of the probe without introducing other organic solvents is more environmentally friendly. Hence, we chose water as the dispersing solution of the probe.

**Figure 5:**
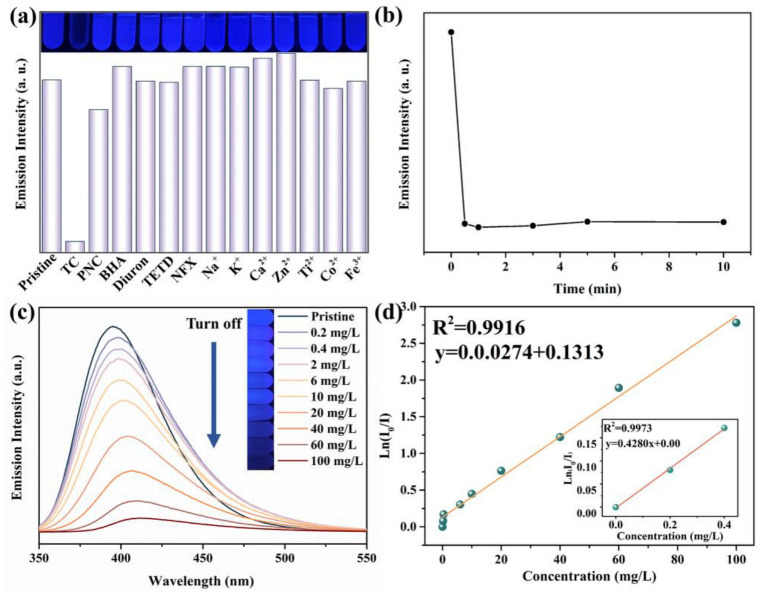
Fluorescence response performances of the H_4_TCPB·4DMF aqueous suspension for TC: (**a**) changes in the emission intensity determined by fluorescence spectra upon the addition of different substances (100 mg/L); the inset is the optical photographs under UV light, λ = 356 nm; (**b**) changes in emission intensity with time when TC is added, C_0_ = 100 mg/L; (**c**) fluorescence spectra with different TC concentrations; the inset is the color change observed in the optical photographs under UV light, λ = 356 nm; (**d**) linear relationship between Ln(I_0_/I) and TC concentration in the range of 0.4–100 mg/L; the inset is the linear relationship between Ln(I_0_/I) and TC concentration in the range of 0–0.4 mg/L.

The fluorescence behavior of the H_4_TCPB·4DMF nanofluorescence probe was investigated with the presence of some common chemicals and metal ions in TC environments, such as TC, penicillin sodium (PNC), salicylic acid (BHA), norfloxacin (NFX), diuron, disulfiram (TETD), Fe^3+^, Co^2+^, Ca^2+^, Na^+^, Ti^4+^, Zn^2+^, and K^+^. As shown in Figure 5a, the fluorescence intensity of H_4_TCPB·4DMF is quenched if TC is contained in the water. However, there is no significant change in fluorescence intensity and wavelength when adding other chemicals and metal ions into the aqueous solution. The luminescent quenching effect can be quantitatively rationalized using the Stern–Volmer equation:

I_0_/I =1+K_sv_ × [Q]

where K_sv_ is the quenching constant, [Q] is the concentration of the analyte (100 mg/L), and I_0_ and I are the luminescence intensities in the absence and presence of the analyte [31]. The K_sv_ value is determined as 64,413 M^−1^, which is two to four orders of magnitude higher than those of the remaining ions and chemicals (Table 1). The large K_sv_ value reveals the strong quenching effect of TC on the luminescence of H_4_TCPB·4DMF, as well as the high sensitivity of H_4_TCPB·4DMF for TC detection. When the initial concentration of TC is 100 mg/L, the fluorescence intensity of aqueous suspensions of H_4_TCPB·4DMF is quenched in an instant and remains stable after that (Figure 5b), indicating a rapid response time. Subsequently, the functional relation between the fluorescence intensity of H_4_TCPB·4DMF and TC concentration was studied. According to the fluorescence spectra and the optical photographs in Figure 5c, the fluorescence intensity of H_4_TCPB·4DMF gradually decreases as the concentration of TC increases. In addition, the natural logarithm of fluorescence intensity (Ln(I/I_0_)) of H_4_TCPB·4DMF) is linearly dependent on the concentration of TC in the concentration range of 0 to 0.4 mg/L and 0.4 to 100 mg/L with a slope value (S) of 0.428 and 0.0274, respectively (Figure 5d). The linear fitting correlation coefficient (R^2^) reached 0.9916 in the high concentration range, indicating that the fluorescence probe has a broad detection range. Such a good linear relationship, with the linear correlation reaching 0.9973, allows for the accurate quantification of TC over a range from 0 to 0.4 mg/L. The limit of detection (LOD) of H_4_TCPB·4DMF toward TC was calculated to be 12 nM based on 3σ/S (Figure S4), where σ is the standard deviation of the blank solution [21]. The detection limits of tetracycline with similar types of fluorescent probes are listed and compared in Table 2.

### 2.5. Sensing Mechanism Exploration

**Figure 6:**
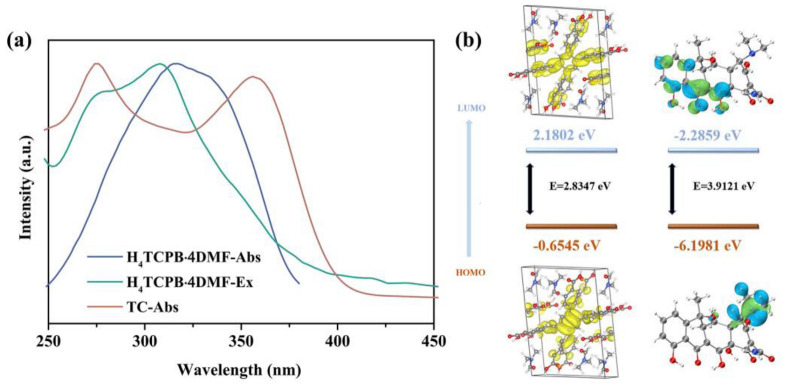
(**a**) UV–vis absorption of H_4_TCPB·4DMF and TC and fluorescence excitation spectrum of H_4_TCPB·4DMF in aqueous solution; (**b**) calculated HOMO and LUMO energies for H_4_TCPB·4DMF and TC.

The mechanism of H_4_TCPB·4DMF sensing TC in aqueous solution was explored. First, the fluorescence excitation spectrum of H_4_TCPB·4DMF, as well as the UV–vis absorption spectra of H_4_TCPB·4DMF and TC, are compared in Figure 6a, and it can be seen that they partly overlap in the wavelength range of 250–400 nm. Moreover, the fluorescence spectrum of H_4_TCPB·4DMF and UV–vis absorption spectrum of TC are shown in Figure S5. It can be seen that there is little overlap between the fluorescence emission of H_4_TCPB·4DMF and the ultraviolet absorption of TC. This rules out the possibility that the fluorescence emission is absorbed, which results in fluorescence quenching. Therefore, the luminescence quenching of H_4_TCPB·4DMF toward TC in the aqueous solution might be attributed to PET and the competition of energy absorption [39]. PET is a process of exciting electron transfer from the photoexcited donor (H_4_TCPB·4DMF) to the lowest unoccupied molecular orbital (LUMO) of the acceptor (TC) [40]. Based on the confirmed crystal structure of H_4_TCPB·4DMF, the DFT calculation was implemented to further prove the PET mechanism. As the diagram in Figure 6b reveals, the HOMO energy levels of H_4_TCPB·4DMF and TC are −0.6545 eV and −6.1981 eV, respectively, while the LUMO energy level of H_4_TCPB·4DMF is 2.1802 eV, which is higher than that of TC (−2.2859 eV). In addition, the functional groups on TC have a strong electron-withdrawing capacity under the combined effect of conjugation and induction [41]. As a result, the luminescence quenching of H_4_TCPB·4DMF in the solution toward TC might be due to the PET process from H_4_TCPB·4DMF to TC. Moreover, the binding of DMF molecules enhances the van der Waals forces around H_4_TCPB, and enhances the photostability and photochemical activities during the process quenching C [42].

## 3. Materials and Methods

### 3.1. Materials

1,2,4,5-Tetrakis (4-carboxyphenyl) benzene (H_4_TCPB, 98%) was supplied by Jilin Chinese Academy of Science-Yanshen Technology Co., Ltd (Changchun, China). Hydrochloric acid (HCl, 36-38%) was purchased from Sinopharm Chemical Reagent Co., Ltd. (Shanghai China) N, N-dimethylformamide (DMF, 99.5%), ethanol (99.7%), methanol (99.5%), acetone (99.5%), tetrahydrofuran (99.5%) nitric acid (HNO_3_, 65%), NaNO_3_ (99%), KNO_3_ (99%), Ca(NO_3_)_2_ (99%), Ti(NO_3_)_4_ (99%), Zn(NO_3_)_2_ (99%), Co(NO_3_)_2_, (99%), and Fe(NO_3_)_3_ (99%) were purchased from Sinopharm Chemical Reagent Co., Ltd. (Shanghai China) A wide range of antibiotics and chemicals, i.e., tetracycline, penicillin sodium, salicylic acid, diuron, disulfiram, and norfloxacin, were purchased from Shanghai Maclin Biochemical Technology Co., Ltd. (Shanghai China).

### 3.2. Analytical Conditions

Powder X-ray diffraction (PXRD) data were collected on an UltimaIV diffractometer from Japan Tokyo. The single-crystal X-ray diffraction (SC-XRD) data collection was carried out on a Bruker AXS D8-VENTURE diffractometer (Mo Kα radiation, λ = 0.71073 Å) (Karlsruhe, Germany). Morphologies were observed using a scanning electron microscope (SEM, JSM-7610F, Tokyo, Japan). Thermogravimetric analysis (TGA) was performed using STA449F5 (Netzsch, Selb Germany). An F-4700 fluorescence spectrophotometer (HITACHI, Tokyo, Japan) was used for the collection of luminescence and excitation spectra. Decay curves and PL quantum yields were collected on an FLS 1000 spectrofluorometer (Edinburgh Instruments, Edinburgh, UK). The ultraviolet-visible (UV–vis) absorption spectra were collected on a UV 2600 (Tianmei, Shanghai, China) spectrophotometer.

### 3.3. Preparation of H_4_TCPB·4DMF

Synthesis of Bulk Crystals: Briefly, 10 mg of H_4_TCPB was dissolved in DMF (1 mL) within a glass vial. Then, the uncapped vial was placed inside a 100 mL beaker containing 10 mL of deionized water. Lastly, the beaker was tightly sealed and allowed to stand at room temperature. After seven days, the colorless sheet crystals of H_4_TCPB·4DMF could be obtained.

Synthesis of Nanocrystals: First, 100 mg of H_4_TCPB was dissolved in 5 mL of DMF. The filtered clear solution was heated at 60 °C for 30 min. Then, 5 mL of H_2_O (room temperature) was added to the above DMF solution dropwise (60 °C). The nanocrystals were separated immediately. The solid products were obtained via centrifugation at 10,000 rpm for 5 min and subsequently purified twice with ethanol.

### 3.4. Fluorescence Measurements

The solid excitation spectra, emission spectra, fluorescence lifetime, and quantum yield of H_4_TCPB·4DMF were collected using Edinburgh FLS1000 (England, Edinburgh). The analytes selected for TC sensing in this work were distributed in H_2_O. The ground powder sample of H_4_TCPB·4DMF (1 mg) was immersed in 10 mL of deionized water and ultrasonicated for about 10 min to form a stable turbid suspension. Spectra were collected immediately after ultrasonic treatment until the suspension was evenly mixed, recording the excitation and emission spectra of the detection system. Then, the H_4_TCPB·4DMF suspension with a concentration of 100 mg/L was used as a fluorescence probe to detect TC in the concentration range of 0.2 mg/L to 100 mg/L at their maximum excitation wavelengths. To study the selectivity ability of H_4_TCPB·4DMF to detect TC, the probe was used for the spectral acquisition of TC. The emission spectra of penicillin sodium (PNC), salicylic acid (BHA), diuron, disulfiram (TETD), norfloxacin (NFX), Na^+^, K^+^, Ca^2+^, Zn^2+^, Ti^4+^, Co^2+^, and Fe^3+^ (100 mg/L) were collected at the maximum excitation wavelength.

### 3.5. Theoretical Simulations

We used first principles to perform all spin-polarized DFT to calculate the orbital energy level of H_4_TCPB·4DMF [43]. The Perdew–Burke–Ernzerhof (PBE) formulation was used for generalized gradient approximation (GGA) [44]. The projected augmented wave (PAW) potential was chosen to describe the ionic core [45,46]. We used a plane-wave basis to take valence electrons into account. The partial occupancy of the Kohn–Sham orbit was facilitated using Gaussian smearing. When the energy changes within a certain range, the electron energy is self-consistent, and the geometric optimization is considered convergent. The Brillouin region is integrated with the Monkhorst–Pack 4×2×2 K-point grid structure. The weak interactions were described using the empirically modified DFT+D3 method in grime format [47].

TC calculations were performed using Gauss 16 software as well as the B3LYP functional and 6-311G(d) basis set. SMD implicit solvation model was used to explain the solvation effect [48,49]. The DFT with gradient corrections and a contracted Gaussian set were used. Bj-damped DFT−D3 was used to improve the calculation accuracy, and the weak interaction was corrected [47]. A Multiwfn software was used to analyze the orbital energy level analysis [50]. The TC track was visualized with VMD-1. 9. 4 software. The specific parameters can be found in the Supporting Information.

## 4. Conclusions

In summary, we successfully assembled a nanocrystalline fluorescent probe of H_4_TCPB·4DMF based on its excellent luminescence properties. It has a strong fluorescence emission and a high quantum yield owing to the highly ordered crystal structure, a large number of hydrogen bonds, as well as π–π, lone pair···π, and lone pair···lone pair interactions. The likely mechanism was elucidated indicating that the PET effect occurs between H_4_TCPB·4DMF and TC, leading to a fluorescence quenching effect. Hence, H_4_TCPB·4DMF nanocrystals are good candidates as nanofluorescence probes for TC. This work enriches the library of crystalline organic fluorescent sensors and provides theoretical support for the design and mechanical studies of organic fluorescent sensors.

## Figures and Tables

**Table 1:** Quenching constants (K_sv_) of various species.

**Name**	TC	PNC	BHA	Diuron	TETD	NFX	
**Ksv**	64,413	738	−101	15	41	−233	
**Name**	Na^+^	K^+^	Ca^+^	Zn^2+^	Fe^3+^	Co^3+^	Ti^4+^
**Ksv**	−17	−27	−45	−86	1	30	4

**Table 2:** A comparison of the limit of detection (LOD) of various fluorescence probes for TC detection in water at room temperature (298 K).

Probe Name	LOD	Ref.
GUCDs	0.165	[32]
N, S-CDs	0.016	[33]
MoS_2_ NPs	0.032	[34]
CDs-Eu^3+^	0.012	[35]
DPA-Ce-GMP-Eu	0.007	[36]
GQDs-Eu^3+^	0.008	[37]
BCDs-Eu/CMP-cit	0.008	[38]
H_4_TCPB·4DMF	0.012	This work

## Data Availability

Not applicable.

## Supplementary Materials

The following supporting information can be downloaded at: https://www.mdpi.com/article/10.3390/molecules28196774/s1, Figure S1: TGA curve of H_4_TCPB·4DMF; Figure S2: Quantum yield measurement of H_4_TCPB·4DMF; Figure S3: Luminescence spectra of TC, which were added into different solutions of H_4_TCPB·4DMF; Figure S4: Emission spectra of deionized water. Figure S5. Fluorescence spectra of H_4_TCPB 4DMF and UV–vis absorption of TC.
