# Supplementary material for: The Fabrication and Mechanism of a Crystalline Organic Fluorescent Probe Based on Photoinduced Electron Transfer
Source: Molecules. 2023 Sep 23;28(19):6774. doi: 10.3390/molecules28196774 (PMC10574209; doi:10.3390/molecules28196774)
Supplement: Supplementary file 1 [file molecules-28-06774-s001.zip › molecules-2585718-supplementary.pdf]

The Fabrication and Mechanism of a Crystalline Organic Fluorescent Probe Based on Photoinduced Electron Transfer

Xinxin Zhang [1], Wei Liu [2], Mei Yang [1] and Zhongyue Li [1,*]

[1] School of Environmental and Material Engineering, Yantai University, Yantai 264005, China; zhangxinxin@s.ytu.edu.cn (X.Z.); meiyang@ytu.edu.cn (M.Y.)

[2] School of Mechanical & Electrical Engineering, Henan University of Technology, Zhengzhou 450001, China; weiliuww@163.com

Electronic Supplementary Information (ESI)

1. Experimental method

1.1. Theoretical simulations detail

In the process of calculating $H_4TCPB{\cdot}4DMF$. We used a plane-wave basis set with a kinetic energy cutoff of 500 eV to take valence electrons into account. The Kohn-Sham orbit was allowed to be partially occupied using a Gaussian smudge with a width of 0.05 eV. When the energy change is less than 10^{-5} eV, the electron energy is considered self-consistent. The geometric optimization is considered convergent when the energy change is less than 0.02 eV A $^{-1}$.

2. Figures

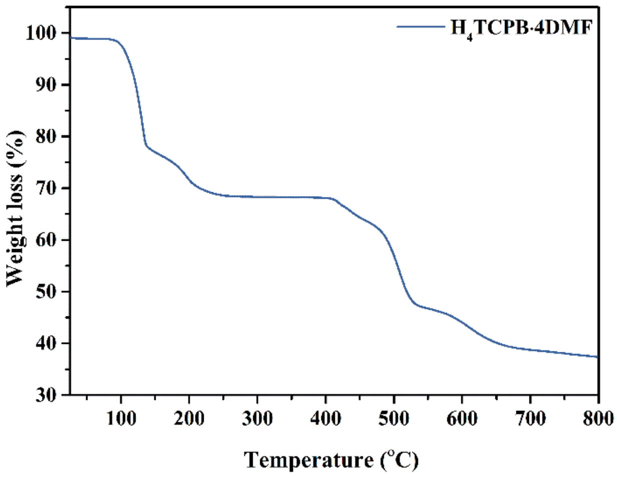

Figure S1. TGA curve of $H_4TCPB{\cdot}4DMF$.

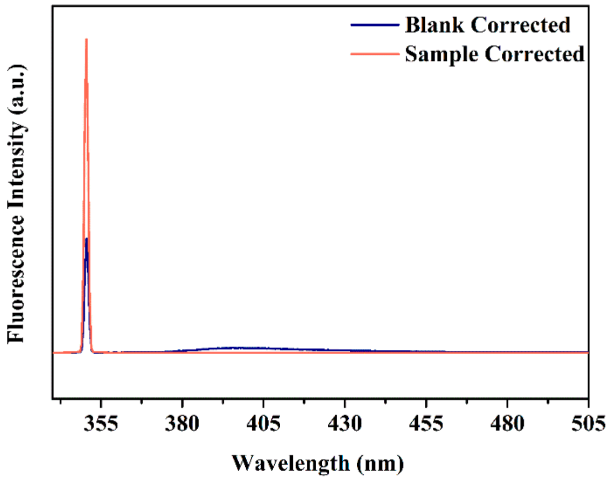

Figure S2. Quantum yield measurement of H4TCPB·4DMF

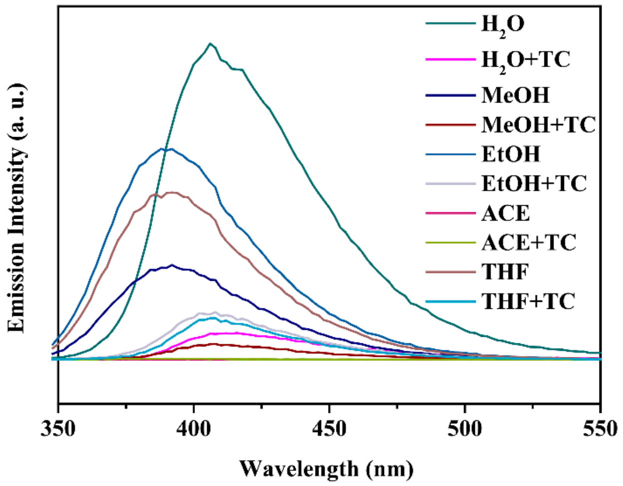

Figure S3. Luminescence spectra of TC were added into different solutions of $H_4TCPB·4DMF$.

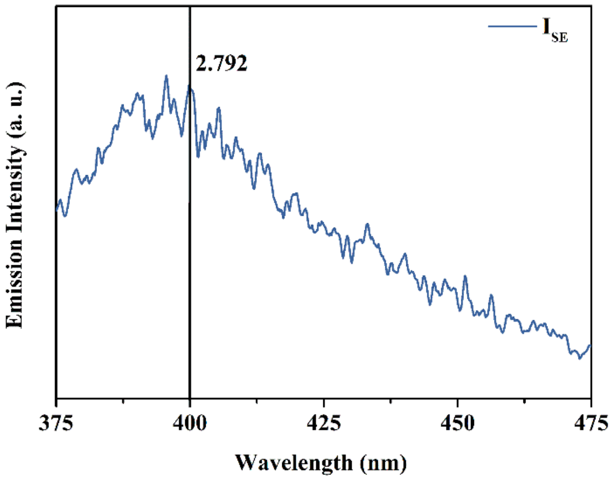

Figure S4. Emission spectra of deionized water.

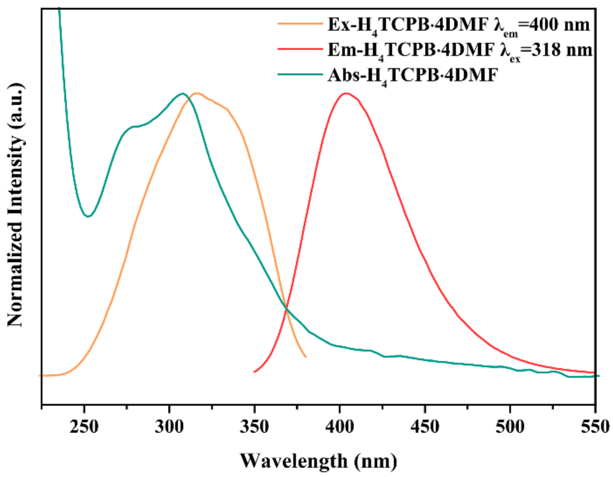

Figure S5. Fluorescence spectra of $H_4TCPB{\cdot}4DMF$ and UV-vis absorption of TC.
